# Zebrafish tracking using YOLOv2 and Kalman filter

**DOI:** 10.1038/s41598-021-81997-9

**Published:** 2021-02-05

**Authors:** Marta de Oliveira Barreiros, Diego de Oliveira Dantas, Luís Claudio de Oliveira Silva, Sidarta Ribeiro, Allan Kardec Barros

**Affiliations:** 1 Department of Electrical Engineering, Laboratory for Biological Information Processing (PIB), Federal University of Maranhão (UFMA), Av. dos Portugueses, 1966, Vila Bacanga, São Luís, MA 65080-805 Brazil; 2 Department of Computational Engineering, Federal University of Maranhão (UFMA), Av. dos Portugueses, 1966, Vila Bacanga, São Luís, MA Brazil; 3 Brain Institute, Federal University of Rio Grande do Norte (UFRN), Av. Sen. Salgado Filho, 3000 Candelária, Natal, RN Brazil

**Keywords:** Engineering, Electrical and electronic engineering, Mathematics and computing, Computational science, Computer science, Learning algorithms

## Abstract

Fish show rapid movements in various behavioral activities or associated with the presence of food. However, in periods of rapid movement, the rate at which occlusion occurs among the fish is quite high, causing inconsistency in the detection and tracking of fish, hindering the fish's identity and behavioral trajectory over a long period of time. Although some algorithms have been proposed to solve these problems, most of their applications were made in groups of fish that swim in shallow water and calm behavior, with few sudden movements. To solve these problems, a convolutional network of object recognition, YOLOv2, was used to delimit the region of the fish heads to optimize individual fish detection. In the tracking phase, the Kalman filter was used to estimate the best state of the fish's head position in each frame and, subsequently, the trajectories of each fish were connected among the frames. The results of the algorithm show adequate performances in the trajectories of groups of zebrafish that exhibited rapid movements.

## Introduction

Social and collective influence is a major challenge for contemporary science, being important for advances in several fields, such as in the organization and in the exchange of information^1^. In groups of animals, behavior has been extensively studied to assess communication among members of the group and obtain a good performance of tasks together. In this sense, there is a growing interest among researchers to assess the collective behavior of animals in order to explain their cognitive evolution^2^. Given this perspective, many researches were based on animal behavior, as well as the creation of bioinspired algorithms for solving optimization problems^3,4^ and behavioral assessment systems ^5–8^, employed in several areas of knowledge. Among the computer vision algorithms for assessing behavior, the methods for tracking objects are the most commonly used, as they allow a thorough assessment of the unexpected movements of different groups of animals, essential for the analysis of collective behavior ^9–17^.

Zebrafish (*Danio rerio*) are widely adopted as a study model in biology ^9–11,17–20^, their school of fish can represent different systems of communication and behavior. In addition, to study the individual behavior of fish in detail, tracking various objects is the most appropriate way. Most tracking systems are based on deep learning^19^, particle filters^21^, adaptive filters^9,10^ and others ^11–13,18,19,22^. However, to be able to track the behavior of fish for a long time, with minimal loss of identity throughout the frames, it is necessary to implement more complex systems that demand higher computational cost. Recently, a tracking system, idtracker.ai, was implemented with two convolutional networks simultaneously to improve the efficiency of animal tracking: one network used to detect when animals touch or cross each other, making the necessary correction, and another one to identify each animal. Thus, groups of zebrafish of up to 100 fish were tracked with an accuracy greater than 99.95%, in an environment with little noise^2^.

Usually, the analysis is based on capturing frames in videos, processing the frame images for detection and tracking of the fish school. However, some technical problems are recurrent when the evaluation of the school of fish is done automatically, with a minimum of human interference. In this regard, many difficulties can be pointed out in data processing in the following steps: detection and tracking. In detection, it is common to have problems in the automatic identification of fish, and when the barycenter is used for detection, there may be limitations in detecting individuals completely; in addition, the failure to detect can exist in times of occlusion or when the visual structure of the fish changes with sudden movements in the tank. On the other hand, tracking, which is a detection-dependent step, can present difficulties over time, where tracking can be lost due to complex fish swimming, detection errors, trajectory fragmentation and, consequently, the loss of fish identity^9,10^.

Several authors have proposed systems to solve these challenges, where they have created complex models of object identification, color tag detection systems^14^, object identification tags^23^, set of variables for unique identification, time complexity algorithms in contour identification^24^ and, detection by separating the head and body of the fish^9,12,20^. However, these algorithms have a high complexity of analysis and processing, which requires greater amounts of frames per second when recording videos (about 60 to 100 frames per second)^9^, and sometimes they are semi-automatic^25^, requiring manual intervention to reconnect lost tracking.

Therefore, it is important that new algorithms minimize several of the problems mentioned above. In this sense, here is proposed a method using YOLOv2 convolutional nets^26,27^, for delimiting the region of the fish head for optimized individual fish detection and Kalman filter for tracking multiple fish in adverse situations, solving problems such as: detection and continuous identification of fish during periods of fast swimming, analysis in low image resolutions, occlusions and minimum number of frames per second, thus showing precision in the trajectory of different quantities of fish.

## Materials and methods

### Ethics statement

All experimental procedures with animals were in compliance and approved by the Ethics Committee on Animal Use (CEUA) of the Federal University of Maranhão (UFMA), campus of São Luís—MA, Brazil (Protocol no. 23115.006194/2018-01). And the procedures were carried out in a way to minimize the animals’ suffering.

### Animals

Zebrafish (*Danio rerio*), adults, of both sexes (n = 13), were obtained from a local pet store (São Luiz—MA, Brazil) and housed in a 22.5 L, 55 cm × 25 cm × 20 cm (length  ×  width  ×  depth). The fish were kept in a closed system with filtered water for two weeks before filming. The water quality was monitored weekly, maintaining the temperature at 26 ± 1 °C and a pH control of approximately 7.2, with mechanical, biological and chemical filtration. The fish were fed four times a day with commercial foods (38% protein, 4% lipids, Nutricom Pet). The lighting was adjusted in a 12/12 h light/dark cycle.

### Experimental apparatus

**Figure 1:**
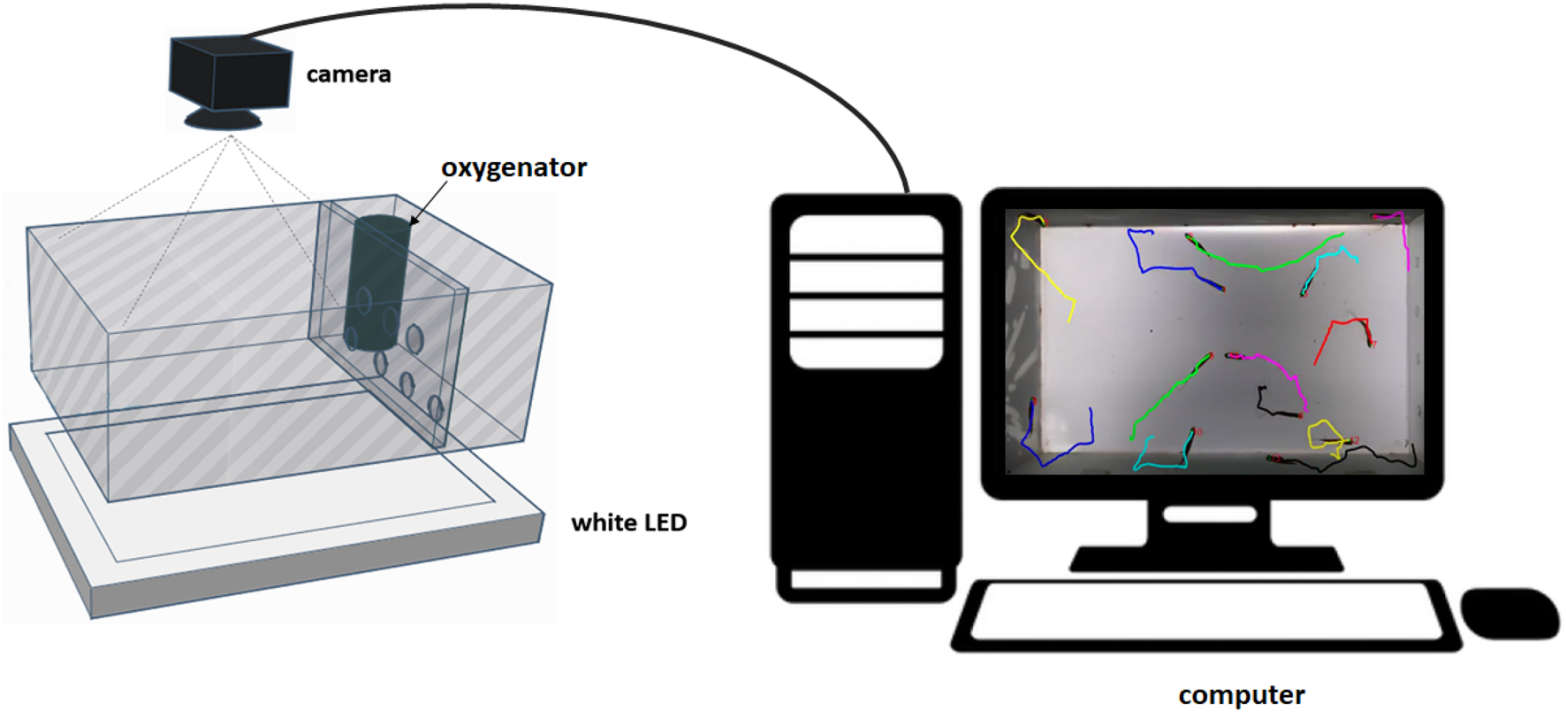
Experiment setup. The groups of zebrafish swam in a sandblasted glass tank (left), placed horizontally above a white LED panel. A camera was mounted above the tank. The images were captured and processed by the computer (right).

To assess the performance of the proposed tracking algorithm, several videos of varying lengths of different groups of fish were captured. The fish normally swam in the 45 cm × 25 cm × 20 cm (length × width × depth), another 10 cm was reserved for oxygenation, with water 10 cm deep, in a sandblasted glass tank on the external walls of the aquarium, avoiding the fish mirroring effect in the detection process in the tracking algorithm. The tank was placed horizontally above a flat light source of white LEDs (60 × 30 cm). The light source was placed at the bottom of the water tank because, this way, the camera is able to capture backlit images and, the object's body (fish) being darker, without many texture resources, facilitates tracking. A full HD camera (C920 Logitech camera), up to 1920 × 1080 pixels, at 30 frames per second was mounted about 50 cm above the tank, the image plane being almost parallel to the water surface. The experiment setup is shown in Fig. 1.

### Proposed system

**Figure 2:**
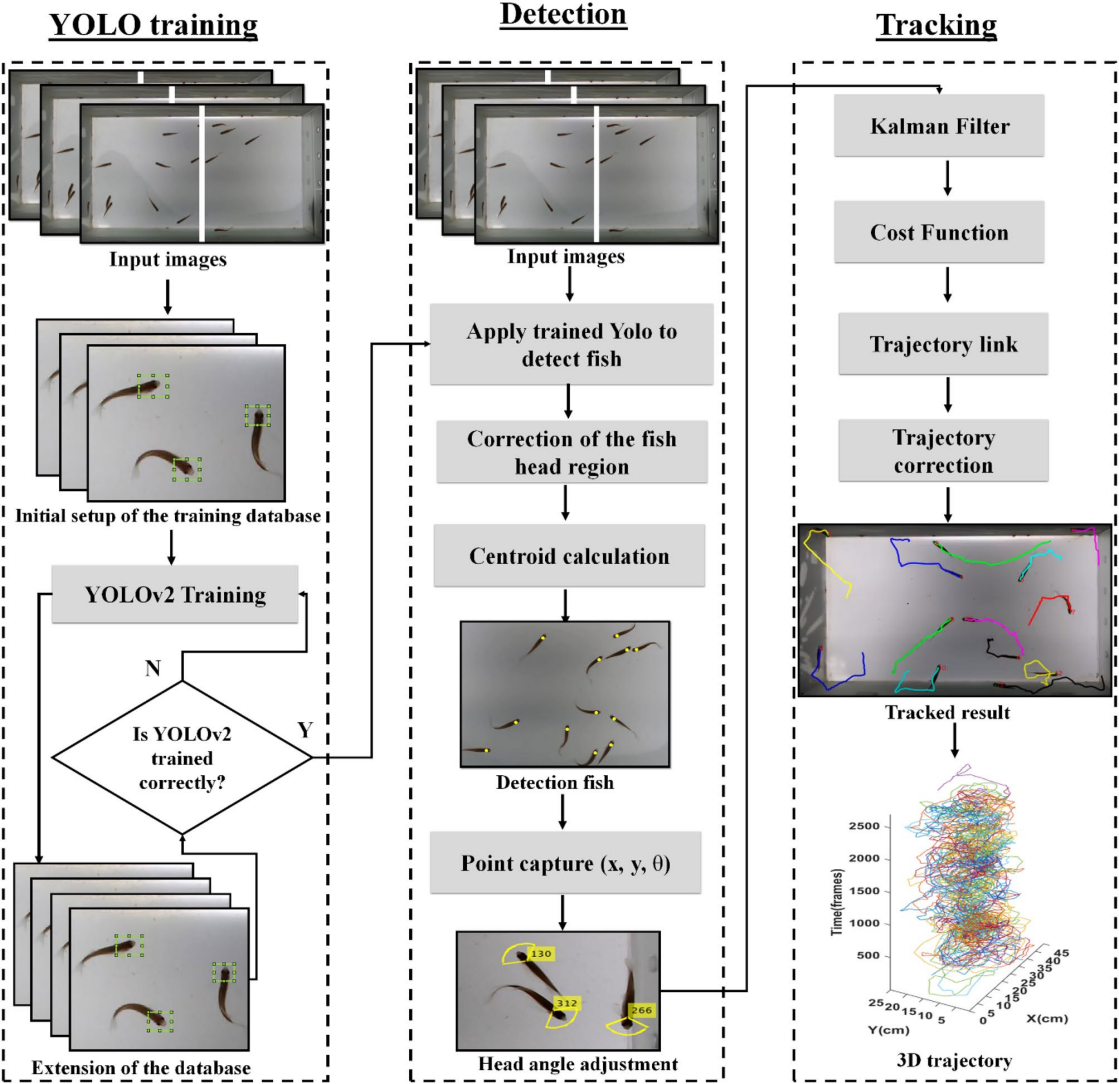
Flowchart of the system proposed for zebrafish tracking. The proposed system is divided into three main stages: delimitation of the fish head, detection and tracking of the fish. The first stage delimits the region of the fish head, used in training the YOLOv2 network to find the next regions in all frames, creating an extended database with all the delimitations of each fish head in the frame. In the second stage, the user’s input images or test data are applied to the trained network for automatic delimitation of the fish head region, then a correction is made to the regions found, if duplicated, and then the centroid is calculated to find the position of the fish in the frame. The head angle is also found at this stage to adjust the Kalman filter; and in the last step, the Kalman Filter is used to predict the fish's next state, adjusting the cost function. Trajectories are linked and corrected to generate a complete trajectory for each fish.

Figure 2 describes the proposed system for tracking each fish in a group, consisting of three stages: delimiting of the fish head region; detection; and tracking of the individual fish to trace trajectories.

In the first step, a set of images with delimitations of the region of each fish head in each frame is used for the initial training of the YOLOv2 network, then the process of marking the other regions of the fish head is automated in the next frames. If the fish head region is correctly delimited, the database can be extended and used as a training base for the convolutional network, otherwise the correction of the delimitation is done in the frames. In the second stage, new frames are given as an entrance to the convolutional network already trained in the previous stage. At this stage, the user enters the video into the system so that the fish detection and tracking process is fully automated, without the need for new markings on the fish head region, because with the trained network there is no need for new markings on the next entry videos. Thus, the boundaries of the fish head region in the new frames of the input video are defined by the YOLOv2 network trained in the first stage of the system, after which the output images are restored to the size of the input image. In this montage, parts of the images overlap, which results in duplications in the detection of fish, these duplications are eliminated, using grid intersection. From the boundaries of the fish head, the centroid of that region is calculated, being the position of the center of the fish head. In addition to the centroid, an ellipse is calculated by Moments, defined to determine the angle of the fish's head, used in the next step.

In the third stage, a Kalman filter is used to estimate the fish's next state, using the head angle for direction. From the estimated values, a cost function is minimized, allowing the creation of trajectories for each fish. It is possible that in the estimation of the state of the fish, there are flaws and, consequently, the trajectory is lost, thus, the links of the trajectories are modeled to connect the fragmented paths, achieving a correct trajectory of the fish , that is, the reconnection of the lost path with the next started path, measured by the shortest distance. From these steps, it is possible to measure the behavioral levels of the fish individually, such as speed, distance covered and agitation of the school.

### YOLOv2 network

The YOLOv2 convolutional network is the second version of YOLO^27^. This network is capable of detecting objects with higher performance than conventional detection methods. The YOLOv2 network can be executed in different sizes, employing a combination of various techniques and training at various scales^26,27^. In addition, it provides good accuracy and speed of execution in relation to other convolutional networks^27^. For the operation of the YOLOv2 algorithm, the image is divided into multiples grids that detect an object inside the grid, making the candidate’s bounding boxes have the same density as the axes of the detected objects. Each grid contains initial bounding boxes with different parameters and a confidence index from a convolution. This index is the probability of the object falling into the bounded box. Then, YOLOv2 tries to eliminate, as much as possible, the bounding boxes that do not correspond to the class of the object, facilitating the learning of the network^26,28^.

After the delineations of the fish head regions are marked by the YOLOv2 network, a grid assembly process is established to form the complete image, using overlapping edges of the images. In this process, there may be objects located at the edges of the image and, consequently, the delimitations of the region of the fish head can be duplicated. To resolve this duplication, the minimum intersection method is used, calculated by,

1 $$Min= \frac{area(AB)}{min(area\left(A\right), area\left(B\right))} ,$$

where A and B represent the bounding boxes for object detection. The result is a defined score value that will pass a decision threshold, the choice, which will limit the detection of an object.

The YOLOv2 network is composed of layers of convolution: Max Pooling, Relu, Batch Normalization and Anchor boxes. In the convolution layer, the backpropagation algorithm trains convolution core weights, shared by the entire image with the same parameters. Relu is a layer that uses an activation function to restrict the network output in a non-linear way from the decision function and the general network, in addition to increasing the speed of the network training. Max Pooling serves to discretize the image, performing non-linear sampling on the interlayer image, dividing it into sets of non-overlapping regions. Batch normalization serves to increase improvements in convergence, without the need for other improvement methods. Finally, in anchor boxes is used to predict the coordinates of the bounding boxes of the fish head region.

### Centroid detection

The fish head region delimitation method is used to obtain information from the center of the fish head; right after the YOLOv2 network finds the correct regions, removing duplicates. Thus, the central position of a given region, corresponding to the centroid of the moving fish, found according to,

2 $$\left\{\begin{array}{c}{M}_{00}= \sum_{i=0}^{w}\sum_{j=0}^{h}R\left(i,j\right)\\ {M}_{10}= \sum_{i=0}^{w}\sum_{j=0}^{h}i x R\left(i,j\right)\\ {M}_{01}= \sum_{i=0}^{w}\sum_{j=0}^{h}j x R\left(i,j\right),\end{array}\right.$$

where, ${M}_{00}$ represents the zero order moment of the fish head region; ${M}_{10}$ and ${M}_{01}$ represent the moment of the first order; and R is a grayscale image of size *w x h* and location bounded by the YOLOv2 detection of an image used as an input, where w is the length and h is the pixel width of the bounding box found in the detection of YOLOv2.

The coordinates of the centroid position $({c}_{{x}_{k}}, {c}_{{y}_{k}})$ of the k-th detected fish head are shown in,

3 $$\left\{\begin{array}{c}{c}_{{x}_{k}}= \frac{{M}_{10}}{{M}_{00}}\\ {c}_{{y}_{k}}= \frac{{M}_{01}}{{M}_{00}}.\end{array}\right.$$

### Motion direction detection

**Figure 3:**
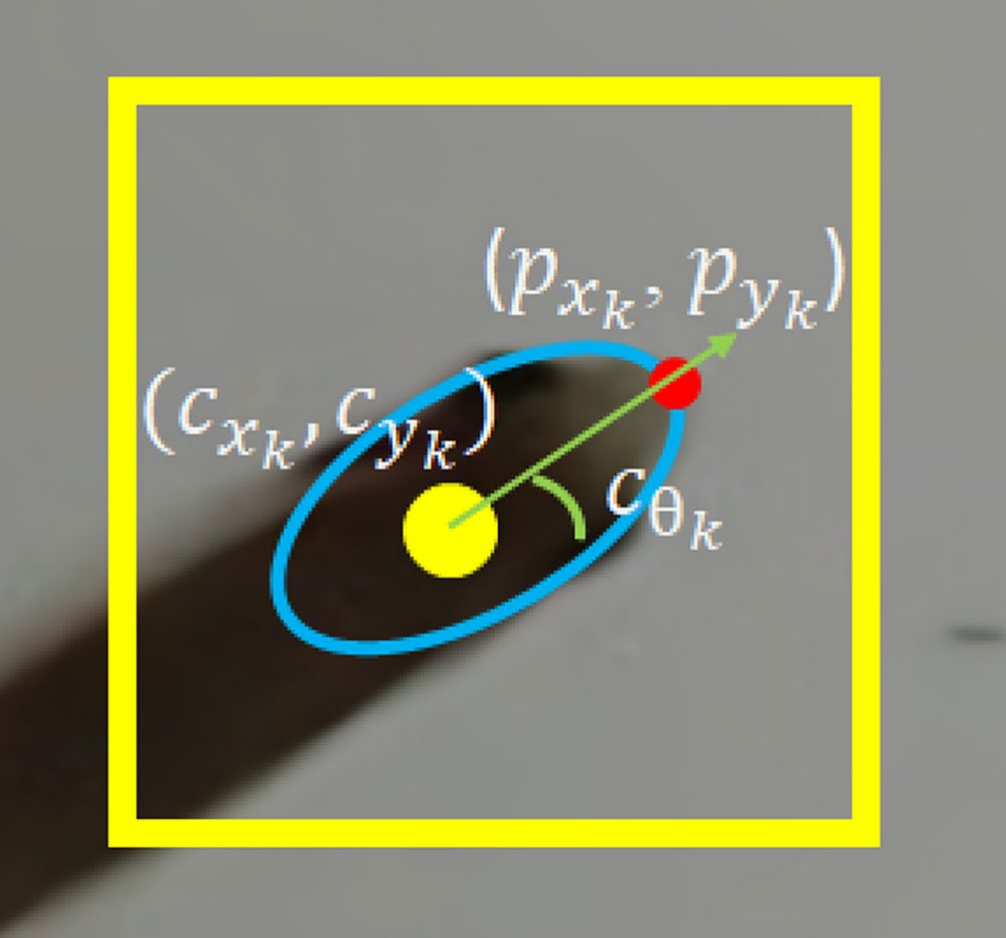
The bounding box is represented by the yellow frame. The centroid position (${c}_{{x}_{k}}, {c}_{{y}_{k}}$) is represented by the yellow dot. The end of the head position (${p}_{{x}_{k}}$, ${p}_{{x}_{k}}$) is represented by the red dot. The direction of movement of the fish is represented by the green arrow that has an angle equal to ${c}_{{\uptheta }_{k}}$.

The direction of the fish's movement is related to the ellipse adjusted on the fish's head. The direction of movement is ${c}_{{\uptheta }_{k}}$, with a range of [0, 2π]. Equation (4) is used to find the fish movement orientation angle.

4 $$c_{\theta_{k}}=\left\{\begin{array}{cc}
\arctan \frac{p_{y_{k}}-c_{y_{k}}}{p_{x_{k}}-c_{x_{k}}}, & p_{x_{k}}>c_{x_{k}} \\
\arctan \frac{p_{y_{k}}-c_{y_{k}}}{p_{x_{k}}-c_{x_{k}}}+\pi, & p_{x_{k}}<c_{x_{k}} \text { and } p_{y_{k}} \geq c_{y_{k}} \\
\arctan \frac{p_{y_{k}}-c_{y_{k}}}{p_{x_{k}}-c_{x_{k}}}-\pi, & p_{x_{k}}<c_{x_{k}} \text { and } p_{y_{k}}<c_{y_{k}} \\
\frac{\pi}{2}, & p_{x_{k}}=c_{x_{k}} \text { and } p_{y_{k}}>c_{y_{k}} \\
-\frac{\pi}{2}, & p_{x_{k}}=c_{x_{k}} \text { and } p_{y_{k}}<c_{y_{k}}
\end{array}\right.$$

where, ${p}_{{x}_{k}}$ and ${p}_{{y}_{k}}$ , respectively, represent the *x* and *y* position of the extremity of the k-th fish head detected (Fig. 3).

### Tracking method

To accurately estimate the movement of the fish, the Kalman filter was used to perform the tracking task. Because the movement of the fish is considered to be a uniform linear motion from one frame to the other, the system can be approximated as a linear dynamic model^9,10,17^. The algorithm settings start with the status vector of the fish's location and orientation. The coordinates of the k-th fish head detected in the frame $t$ are indicated as $({c}_{{x}_{k},t},{c}_{{y}_{k},t},{c}_{{\uptheta }_{k},t})$, therefore, the state vector ${x}_{t}$ is defined as $[{c}_{{x}_{k},t},{c}_{{y}_{k},t},{c}_{{\uptheta }_{k},t}{,{\dot{c}}_{{x}_{k},t},{\dot{c}}_{{y}_{k},t},{\dot{c}}_{{\uptheta }_{k},t}]}^{T}$. The state and observation equation in the Kalman filter can be described as:

5 $$\left\{\begin{array}{c}{x}_{t}={Fx}_{t-1}+ {w}_{t}\\ {z}_{t}={Hx}_{t}+ {v}_{t}\end{array}\right.,$$

where the $F$ e $H$ are the state transition and observation matrix of the target at i *t*, respectively, ${w}_{t}$ and ${v}_{t}$ are noise. The first step of the Kalman filter is to predict the state vector at instant *t.* The estimate of the state vector ${\widehat{x}}_{t}$ and its error covariance ${\widehat{P}}_{t}$ at instant $t$ can be predicted by:

6 $${\widehat{x}}_{t}={Fx}_{t-1},$$

7 $$\widehat{P}={FP}_{t-1}{F}^{T}+{Q}_{t},$$

8 $$F= \left[\begin{array}{cccccc}1& 0& 0& dt& 0& 0\\ 0& 1& 0& 0& dt& 0\\ 0& 0& 1& 0& 0& dt\\ 0& 0& 0& 1& 0& 0\\ 0& 0& 0& 0& 1& 0\\ 0& 0& 0& 0& 0& 1\end{array}\right],$$

where ${Q}_{t}$ is the noise state covariance matrix ${v}_{t}$, ${P}_{t-1}$ is the error covariance matrix in $t-1$ and $dt$ is the time interval between two frames.

The second stage is the association of the predicted positions of each fish by the Kalman filter with the centroid of the fish, in order to form the trajectory of each fish in the pictures. Thus, the association centroid of the fish $({c}_{{x}_{k}}, {c}_{{y}_{k}})$ must follow the one-to-one criterion, and a tracker must be linked to a maximum of one value, which will be associated with a maximum of one tracker.

When the data association ends, the state vector and the error covariance matrix are updated by,

9 $${x}_{t}={\widehat{x}}_{t}+ {K}_{t}\left({\widehat{z}}_{t}-{HK}_{t}\right),$$

10 $${P}_{t}={(I-K}_{t}H)+{\widehat{P}}_{t},$$

where I is the identity matrix and ${K}_{t}$ is the Kalman gain at instant *t,* calculated as,

11 $${K}_{t}={\widehat{P}}_{t}{H}^{t}(H{\widehat{P}}_{t}{H}^{t}-R{)}^{-1},$$

12 $$R= \left[\begin{array}{ccc}24.1& 0& 0\\ 0& 24.8& 0\\ 0& 0& 22.7\end{array}\right],$$

13 $$H=\left[\begin{array}{cccccc}1& 0& 0& 0& 0& 0\\ 0& 1& 0& 0& 0& 0\\ 0& 0& 1& 0& 0& 0\end{array}\right],$$

where $R$ is the covariance matrix of the observation noise ${v}_{t}.$

### Cost function

The construction of the paths followed by the fish are developed through Muncres' implementation of the Hungrian algorithm^29^. This algorithm makes a direct assignment of m sources to k targets through an $m x k$ matrix called a cost matrix. A matrix ${P }_{\mathrm{m}\times \mathrm{ k}}$, shown in Eq. (14), is created to save the cost of associating source objects $\mathrm{S }= \{{\mathrm{s}}_{1},{\mathrm{s}}_{2},\dots ,{\mathrm{s}}_{m}\}$ to the target objects $\mathrm{R }= \left\{{\mathrm{r}}_{1},{\mathrm{r}}_{2},\dots ,{\mathrm{r}}_{k}\right\}$ in the frame t, where ${\mathrm{s}}_{m}$ is a coordinate of the state variable of the observation variable in frame t-1,$m$ is the number of predicted coordinates, ${\mathrm{r}}_{n}$ is the coordinate variable of the observation,$k$ is the number of detected coordinates.

14 $$P\left({s}_{i},{r}_{j}\right)=\left[\begin{array}{cccc}{f}_{{s}_{1},{r}_{1}}& {f}_{{s}_{1},{r}_{2}}& \cdots & {f}_{{s}_{1},{r}_{n}}\\ {f}_{{s}_{2},{r}_{1}}& {f}_{{s}_{1},{r}_{2}}& \cdots & {f}_{{s}_{1},{r}_{1}}\\ \vdots & \vdots & \vdots & \vdots \\ {f}_{{s}_{m},{r}_{1}}& {f}_{{s}_{m},{r}_{2}}& \cdots & {f}_{{s}_{m},{r}_{n}}\end{array}\right],$$

The element ${f}_{{s}_{i},{r}_{j}}$ in the matrix indicates the cost to connect the i-th predicted coordinate track s to the j-th detect coordinate r. The value of $P\left({s}_{i},{r}_{j}\right)$ is calculated according to Eq. (15)

15 $${f}_{{s}_{i},{r}_{j}}= {w}_{1}\left(\sqrt{({s}_{x}-{{r}_{x})}^{2}+({s}_{y}-{{r}_{j})}^{2}}\right)+{w}_{2}\left(\sqrt{({s}_{x}-{{r}_{x})}^{2}+({s}_{y}-{{r}_{j})}^{2}}\right)\bullet \frac{\left|{s}_{\theta }-{r}_{\theta }\right|}{2\pi } ,$$

where ${w}_{1}$ and ${w}_{2}$ are scalars used to weight each part of the function, ${s}_{x}$,${s}_{y}$ and ${s}_{\theta }$ are, respectively, the coordinate values of the $x, y$ axis and orientation $\theta$ of the fish head detection, ${r}_{x}$,${r}_{y}$ and ${r}_{\theta }$ are respectively the coordinate values of the $x, y$ axis and orientation $\theta$ of the estimated coordinate.

The path assigned for each fish, frame by frame, is performed using the Muncres implementation of the hungrian algorithm, which looks for unique assignments, that is, it assigns the object of the previous frame i to only one target object j in the current frame.

### Reconnection of the trajectory

Some trajectories may be lost over time, due to occlusion of the fish and the corresponding interval between images, causing fragmentation of the trajectory. If the trajectory was incomplete compared to the initial trajectory, before a loss of identification occurred, a second calculation of the trajectory was proposed based on Qian et al., and Wu et al.^10,30^. If no coordinate was associated in $$\beta$$ consecutive frames, then the trajectory is interrupted. Position and time ($${x}_{end},{y}_{end},{t}_{end})$$ trajectory $${\Gamma }_{i}$$ are stored in $${\tau }_{i}$$, where *i* is the trajectory number. If a new coordinate appears without any assignment, its position and time ($${x}_{start},{y}_{start},{t}_{start})$$ trajectory $${\Gamma }_{j}$$ are stored in $${\rho }_{j}$$, where j is the trajectory number. Assuming $${\Gamma }_{i}$$ an interrupted trajectory and $${\Gamma }_{j}$$ a trajectory with an unassigned coordinate, the connection of one trajectory with the other can be performed using the condition of Eq. (16)

16 $$constrain distance: \left\{\begin{array}{c}1 \quad Dist \left({\tau }_{i},{\rho }_{j}\right)<d\\ 0 \quad otherwise\end{array}\right.,$$

17 $$Dist \left({\tau }_{i},{\rho }_{j}\right)= \sqrt{{\left({x}_{end}-{x}_{start}\right)}^{2}+{\left({y}_{end}-{y}_{start}\right)}^{2}},$$

**Figure 4:**
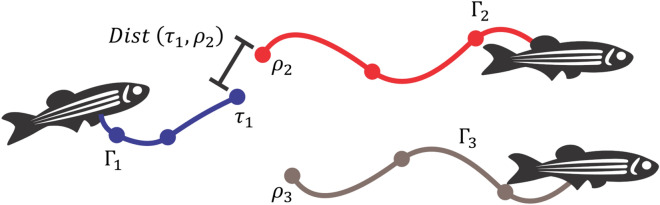
Reconnection of the trajectory of the fish. In this situation there is an interrupted trajectory ${\Gamma }_{1}$ and two new trajectories ${\Gamma }_{2}$ e ${\Gamma }_{3}$. For trajectory connection ${\Gamma }_{1}$ the condition of Eq. (16) is verified with respect to the trajectories ${\Gamma }_{2}$ e ${\Gamma }_{3}$. If the result is true for both trajectories, the trajectory chosen for the connection is the one with the shortest distance defined by Eq. (17). In the figure, the lowest value is $Dist \left({\rho }_{2},{\tau }_{1}\right)$, then their trajectories can be connected.

The condition in Eq. (16) indicates whether the distance between the start position of ${\rho }_{j}$ and end ${\tau }_{i}$ is less than *d*, where *d* is a user-defined distance threshold value, if true trajectories are connected. If there is more than one interrupted trajectory, the condition of Eq. (16) is calculated following the order of the trajectory with the lowest value of ${t}_{end}$ up to the highest ${t}_{end}$. In cases where there is more than one path without attribution, the connection is made with the coordinate ${\rho }_{j}$ that presents a smaller distance between the trajectory ${\tau }_{i}$, given by Eq. (17). Figure 4 illustrates the process of connecting an interrupted trajectory with a new trajectory.

### Data ground truth

To quantitatively evaluate the proposed algorithm, the tracking performance was compared with ground truth. The basic truth data was manually labeled according to the position of the zebrafish movements in each frame. Detection was done using the region of the fish head. For each video recorded, a unique ID was assigned to a fish. If the fish was not marked or there was an occlusion, the fish would be found manually in the evaluation of each frame. A total of 15,106 frames were analyzed individually, in order to guarantee the consistency of the database and a good evaluation for the algorithm.

### Detection evaluation metrics

The detection performance evaluation was measured for all video sequences. A total of 15,106 frames were recorded manually to confirm the performance of the algorithm in the correct identification of the fish, mainly in cases of occlusion. Thus, precision and recovery rate methods were used to assess object detections. Defined in the Eqs. (18) and (19):

18 $$Precision=\frac{True Positive}{True Positive+False Positive}$$

19 $$Recall= \frac{True Positive}{True Positive+False Negative}$$

where the value of true positive is the total number of correct detections in all frames; false negative is the total number of detections lost and false positive is the total of regions detected incorrectly. That way, the metric ${F}_{mensure}$ is the weighted calculation of precision and recall (Eq. 20).

20 $${F}_{measure}= \frac{2 x (Recall x Precision)}{Recall+Precision}$$

In addition, the Similarity Index (SI) was used, which measures the number of correctly detected objects, penalizing undetected objects, whether due to occlusion or detection error. That way, ${Num}_{missed}$ is the number of undetected objects and ${Num}_{GroundTruth}$ is the number of objects detected from the ground truth (Eq. 21).

21 $$Similarity Index= {F}_{measure}-\frac{{Num}_{missed} }{2 x {Num}_{GroundTruth} }$$

To evaluate the proposed method in case of occlusions, the metrics OR (occlusion ratio) and ODR (occlusion detection ratio) were used, presented in the Eqs. (22) and (23):

22 $$OR= \frac{Total Number Of Occlusions}{Total Number Of Targets}$$

23 $$ODR= \frac{Successful Number Of Occlusions Detection}{Total Number Of Oclusions}$$

The evaluation of detection’s performance was based on the error rate of undetected fish (miss ratio) and the error rate of erroneous detection (error ratio), in which they are applied to evaluate the performance of the detection stage, calculated as the Eqs. (24) and (25),

24 $$MissRatio= \frac{Total Number Of Undetected Fish In All Frames}{Number Of Fish \times Number Of Frames}$$

25 $$ErrorRatio= \frac{Total Number Of Wrongly Detected Fish In All Frames}{Number Of Fish \times Number Of Frames}$$

### Tracking evaluation metrics

The tracking performance of the proposed system was assessed using the Correct Tracking Rate Index (CTR), which describes the percentage of frames correctly tracked for a single fish, such as Eq. 26:

26 $$CTR= \frac{\sum (Number Of Correct Frames Of Single Fish)}{Number Of Fish \times Number Of Frames}$$

And the correct identification reason (CIR) that represents the probability of correct identification of all fish after an occlusion, such as Eq. (27):

27 $$CIR= \frac{Times That All Fish Get Correct Identity After Occlusion}{Number Of Oclusions Events}$$

## Results

### Video data set for analysis

To test the proposed system, we used benchmarks containing eight video sequences, six of which are video sequences related to our own experiment (D1–D6, see videos S1–S6), having a total of 8427 frames with 1920 × 1080 resolution; and two sequences of videos available from previous publications, being D7 and D8 (Romero-Ferrero et al.^2^; see videos S7 and S8) with a total of 6820 frames (3712 × 3712 resolution). All data sets were recorded at 30 frames. Tracking performance was compared to ground truth data. The videos had different durations and number of fish, ranging from 3 to 100 fish, and are described on Table 1. The experimental apparatus is shown in Fig. 1. The behavior of the fish was measured by the average speed, showing two conditions of movement: slow and fast (feeding period). The proposed tracking algorithm was developed using a personal computer (Intel Core i7 7700HQ CPU at 2800 8 GB RAM, Geforce gtx 1050 4 GB off-board graphics card) using Matlab R2019b software.

**Table 1:** List of videos used for zebrafish tracking.

Dataset	Fish	Frames	FPS	Resolution	Average speed per fish (cm/s)
D1	3	904	30	1920 × 1080	9.42
D2	6	1,269	30	1920 × 1080	7.81
D3	8	789	30	1920 × 1080	6.37
D4	10	1,366	30	1920 × 1080	6.44
D5	13	2,422	30	1920 × 1080	7.44
D6_food	13	1,519	30	1920 × 1080	10.22
D7 (idtracker.ai ^2^)	10	5,410	30	3712 × 3712	8.14
D8 (idtracker.ai ^2^)	100	1,410	30	3712 × 3712	7.49

*FPS* frames per second.

### YOLOv2 network architecture

**Figure 5:**
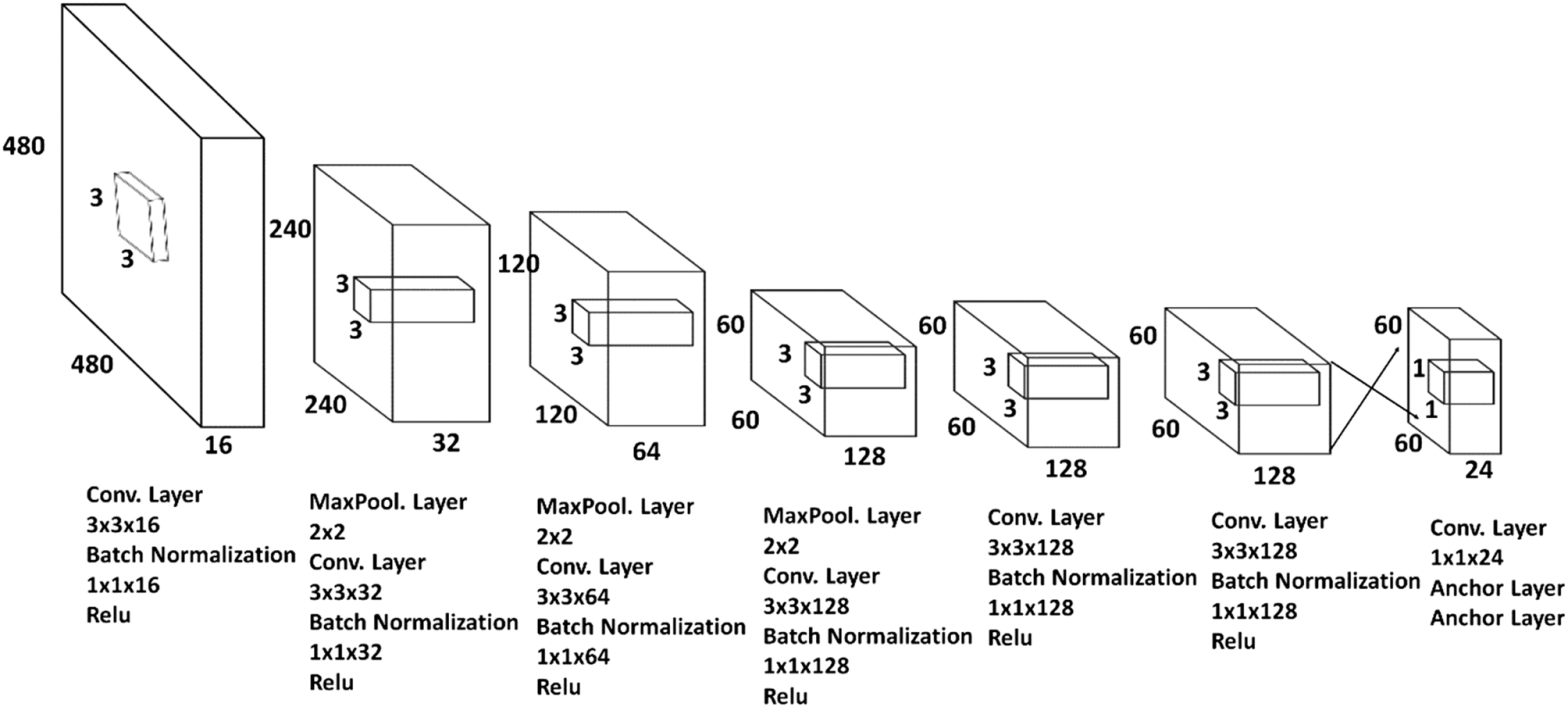
Architecture of the proposed YOLOv2 network framework. The input image has a size of 480 × 480, and each layer is reduced by filters of up to 60 × 60. All combinations of the network architecture blocks have a convolution layer, Batch normalization and Relu, with the exception of the last layer that presents the exit through convolution and the anchor boxes. Only the three initial layers have Max pooling.

The architecture of the YOLOv2 network used in this article is shown in Fig. 5. In this architecture, there are 24 layers, seven layers of convolution, six layers of Relu, three layers of Max Pooling, six layers of Batch normalization and two layers of anchoring. The input layer corresponds to the network input, with the image size of 480 × 480 × 3. YOLO's convolutional layers decrease the sample by a factor of 32, obtaining the delimitations of the region of the fish head, with the convolution filter size being a 3 × 3 block and the convolution step is 1. The Max Pooling layer has the size 2 × 2, with step 2 and filling 0. All main blocks have a Relu operation layer. At the end of the block, it has two layers of anchor boxes: one for transformation and one for output. The first anchor layer transforms the output of the raw convolutional network into a format necessary to produce object detections; and the second defines the parameters of the anchor box, implementing the loss function used to train the detector^31^. In this way, YOLOv2 will be able to make use of the lower and upper level information, increasing the precision of the detection and location of the region of the fish head in the image.

### YOLOv2 network training

During the training, a set of 3200 image grids, 1100 image grids of videos from our experiment and 500 image grids from the database of Romero-Ferrero et al^2^ (approximately 1600 original size images) were initially manually labeled with the region of the fish head, being fed into the YOLOv2 network.

After the initial training, the YOLOv2 network was used to label and create the boundaries on the fish heads in new images, forming an automatic labeling process. To validate the training, a test base was established to know the assertiveness of the labeling of fish from the initial base, in case of wrong or missing labels. To improve the result of the initial training, the training base was extended with new images labeled and marked from the automatic process, but with manual corrections in the event of any error. This process reduced the time to create a training base. When the training was able to create all the delimitations of the region of the fish head correctly, the training base used was sent to the YOLOv2 network for official training.

**Figure 6:**
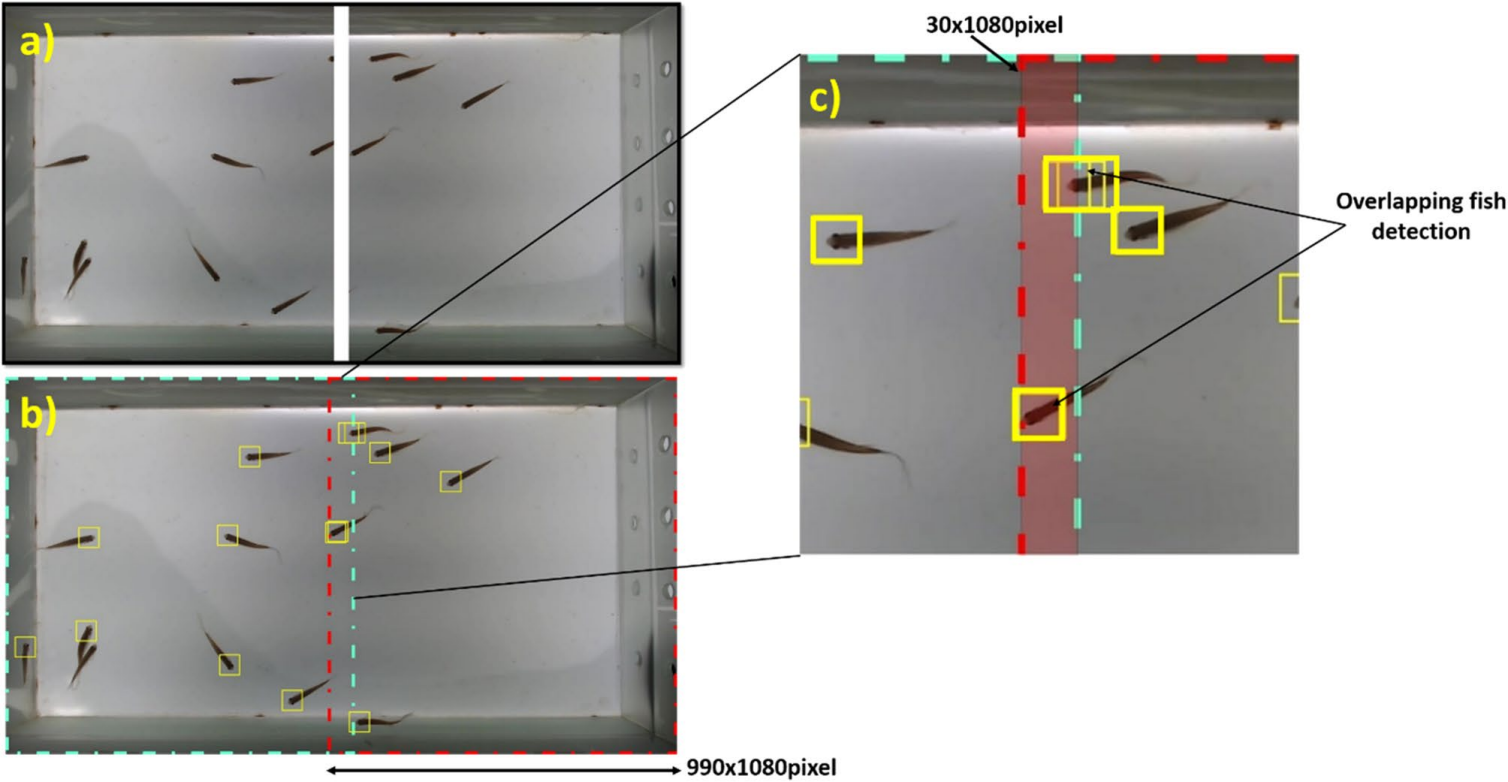
Example of a grid image with overlapping edges and duplication of the delimitation the region of the fish head. **(a)** Input image for YOLOv2 network divided into grids for the process of fish detection. **(b)** Assembly of grids with overlapping edges. **(c)** The red stripes represent the overlapping of the edges of the images during the assembly process (equivalent to 30 pixels wide). Fish were detected in the edge of grids and the correction was made using the grid intersection method presented in Eq. (1).

Because the YOLOv2 networks are trained and evaluated in small resolutions (228 × 228 in the case of resnet50) and the frame size of the proposed system is 1920 × 1080 pixels, the image when resized could lose important features of the region of the fish head. The input images from the YOLOv2 network were cut by dividing the image in half. The reduction is made by creating two grids of 960 × 1080 pixels with an overlap of 30 pixels, totaling an image of 990 × 1080 pixels (Fig. 6). Then each grid is reduced to a dimension of 480 × 480pixels which is used as input to the YOLOv2 network. After the creation of the delimitations of the region of the fish head in the images meshed by the net, the process of assembling the complete image is established, using the image overlay algorithm, and the correction of duplication of the region of the fish head located on the edges of the grids (Fig. 6c). If this result is greater than the 0.4 threshold, then the same detection will be considered and, thus, a single delimitation of the region of the fish head will be assigned to the fish located on the edge of the image. This way, the detection of the delimitation of the region of the fish head can appear at the edge of the grids without loss of data, but with duplication corrected.

The YOLOv2 training protocol was based on MATLAB tools for convolutional networks, following the parameters of the convolution layers. The training process was carried out for 300 periods, using a learning rate of 0.001.

### Detection evaluation

The results of the detection evaluation are presented in Table 2. The proposed method presented a superior performance of up to 0.99 of precision for all data sets in our experiment (D1–D6), thus showing that the proposed detection method based on YOLOv2 to create delimitations of the region of the fish head and assist in the calculation of the centroid, helps in the detection with different quantities of fish in the groups. Evaluating the detection in the videos, in which the fish presented slower movements (D1, D2 and D3, with an average speed of 9.42 cm/s, 7.81 cm/s and 6.44 cm/s, SI: 0.9963, 0.9883 and 0.9884, respectively), with groups of 3, 6, and 8 fish, the precision reached 1.00 and F-measure reached 0.99.

**Table 2:** Performance of the detection evaluation.

Dataset	Occlusions	Precision	Recall	OR	ODR	Miss ratio	Error ratio	F measure	SI
D1	10	1.0000	0.9963	0.0037	0.8000	0.0037	0.0000	0.9982	0.9963
D2	49	1.0000	0.9883	0.0064	1.0000	0.0117	0.0000	0.9941	0.9883
D3	59	1.0000	0.9884	0.0093	1.0000	0.0116	0.0000	0.9942	0.9884
D4	135	0.9999	0.9786	0.0099	0.6519	0.0214	0.0001	0.9891	0.9784
D5	1065	0.9998	0.9677	0.0338	0.5925	0.0323	0.0002	0.9835	0.9673
D6_food	715	0.9987	0.9663	0.0362	0.6252	0.0337	0.0013	0.9822	0.9653
D7 (idtracker.ai^2^)	343	0.9998	0.9989	0.0063	0.7347	0.0011	0.0002	0.9994	0.9988
D8 (idtracker.ai^2^)	399	0.9999	0.9340	0.0028	0.7268	0.0660	0.0001	0.9658	0.9328

**Figure 7:**
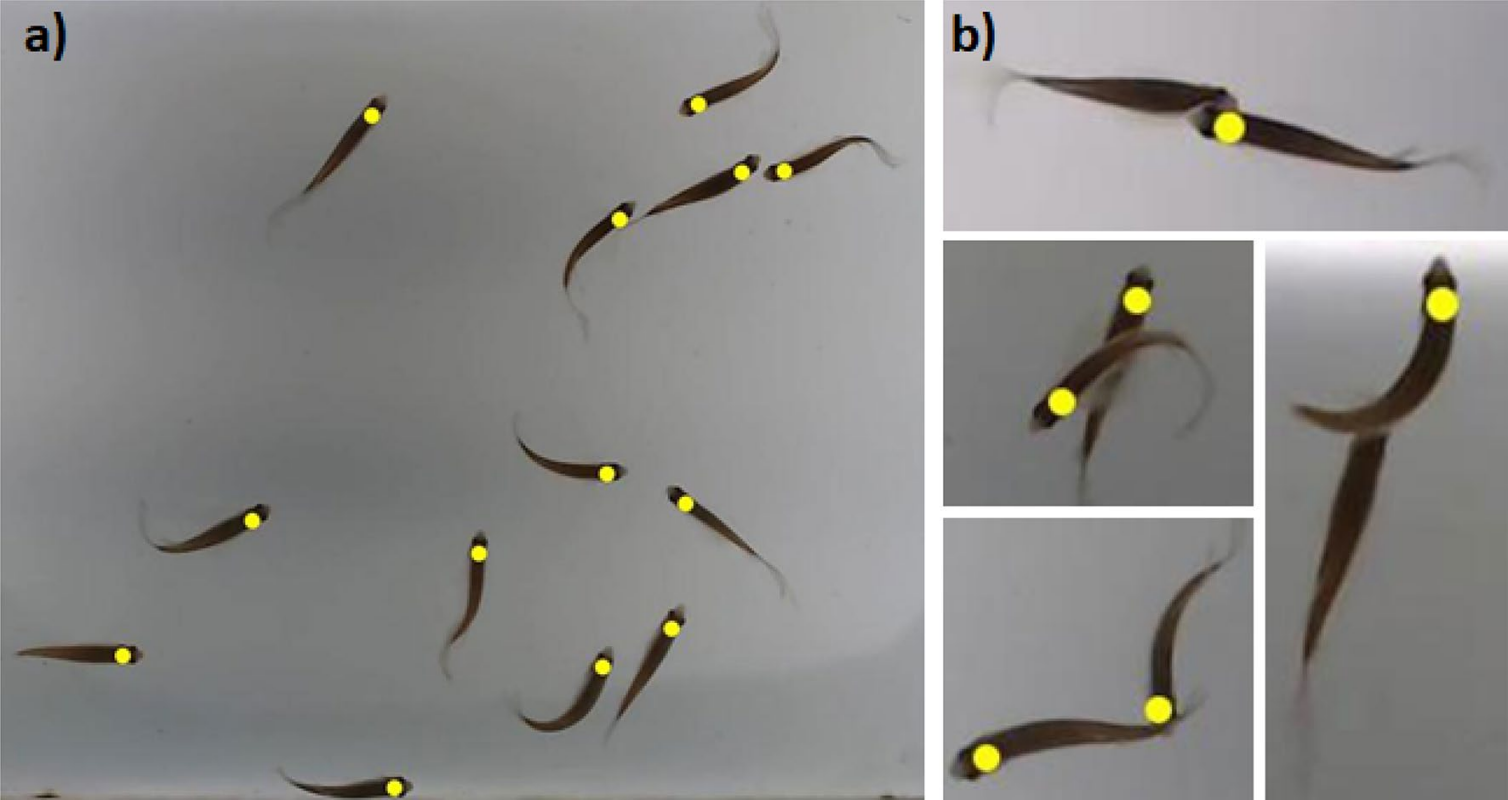
Fish detection. **(a)** Image of the complete detection of the fish head region for a group of 13 fish; **(b)** Examples of fish occlusion events, in some cases, there was a failure in detection.

In faster movements (D6_food), during the feeding period, some losses in the detection were observed, but the algorithm was able to detect fish with good precision (0.9987), even with high occlusion frequency, a total of 715 occlusions (Table 2), and with high average speed per fish about 10.22 cm/s (Table 1). It was noticed that the occlusion is related to the fast and agglomerated swimming of the school, mainly in the feeding period (D6_food, OR: 0.0362, ODR: 0.6252, Miss ratio: 0.0337, Error ratio: 0.0013, F-measure: 0.9822, SI: 0.9653). Figure 7 shows some examples of detection and occlusions.

This method uses the fish head region to calculate the centroid and detect the fish in the images. On the other hand, in other previously published works, the authors used the fish's body as a complement to the detection and identification^9,10,17,32^. Thus, the proposed method was able to detect fish in several situations considered difficult for the detection to remain correct, being incapable only in times of occlusion, where the fish's head was totally occluded by another fish. In addition, there was little loss of detection when the environment was noisy and challenging. Thus, it was possible to evaluate videos in different conditions, so that the method does not limit only one tested video input; it can be used in other shooting conditions.

We tested the proposed algorithm in another dataset with videos containing groups of 10 fish (video D7) and a group of 100 fish (video D8), available for free by Romero-Ferrero et al.^2^, referring to the idtracker.ai system, the videos had high resolution (3712 × 3712 pixels) and different environmental configuration from our experiment. The results of videos D7 and D8 were greater than 0.999 precision for both analyzed videos (Table 2). The fish were at an average speed of 8.14 cm/s and 7.49 cm/s, videos D7 and D8, respectively. The number of occlusions was relatively low, compared to the other videos analyzed in this article, thus obtaining the D7: 343 occlusions, OR: 0.0063, ODR: 0.7347, F-measure: 0.9994 and SI: 0.9988, and D8: 399 occlusions , OR: 0.0027, ODR: 0.7268, F-measure: 0.9658 and SI: 0.9328. It is observed that the increase in the quantity of fish (100 fish) did not interfere with the detection precision of the proposed algorithm.

### Tracking evaluation

Table 3 shows the tracking result, using the CTR and CIR metrics. The tracking is calculated similarly to the detection of a fish, calculating the percentage of the frames correctly tracked for each fish and the correct probability of all fish. The CTR describes the percentage of frames correctly tracked for each fish and, in this method, the exchange of identifications is not evaluated, but only if the tracker can follow the fish head correctly. In this study, the correct tracking of the fish in the frame was defined by the distance from the actual position of the center of the fish head in relation to the center of the tracking at a distance of up to 15 pixels, a greater distance or the absence of a tracking in the frame is considered to be an incorrect tracking. The results show a good percentage of frames correctly tracked of a single fish during a video, reaching up to 100% in the tracking when the quantity of fish is reduced (D1, CIR: 1.00, CTR: 1.00; and D2, CIR: 1.00, CTR: 0.99). It was observed that the fish loses its identification for a short period of time (D3, CIR: 0.95, CTR: 0.99 and D4, CIR: 0.96, CTR: 0.99). It is noticed that larger quantities of fish increase the amount of exchanges or losses of identification. In addition, the probability of correct identification of all fish after an occlusion is compromised with larger quantities of fish in the dataset or even with faster swimming motions (D5, CIR: 0.91, CTR: 0.98 and D6_food : CIR: 0.83, CTR: 0.97).

**Table 3:** Performance of the evaluation tracking.

Dataset	CIR	CTR
D1	1.0000	1.0000
D2	1.0000	0.9970
D3	0.9500	0.9978
D4	0.9629	0.9987
D5	0.9115	0.9887
D6_food	0.8394	0.9795
D7 (idtracker.ai ^2^)	0.9375	0.9969
D8 (idtracker.ai ^2^)	0.9285	0.9983

In this sense, it was already expected that in the feeding period, the tracking of fish would be lost, as there were many occlusions and totally unexpected movement. The biggest tracking losses were in videos D5 and D6_food. The tracking of videos D7 and D8 performed well, obtaining a CTR value of up to 0.99 in tracking fish (D7, CIR: 0.9375, CTR: 0.9970 and D8, CIR: 0.9285 and CTR: 0.9983). It is observed that the algorithm was able to track fish from another dataset, where the videos were recorded with a higher resolution than our images (Table 1) and experimental environment different from that proposed here. In addition, it is notable that the spread of errors decreases as occlusions happen less frequently when the fish swim in the tank.

**Figure 8:**
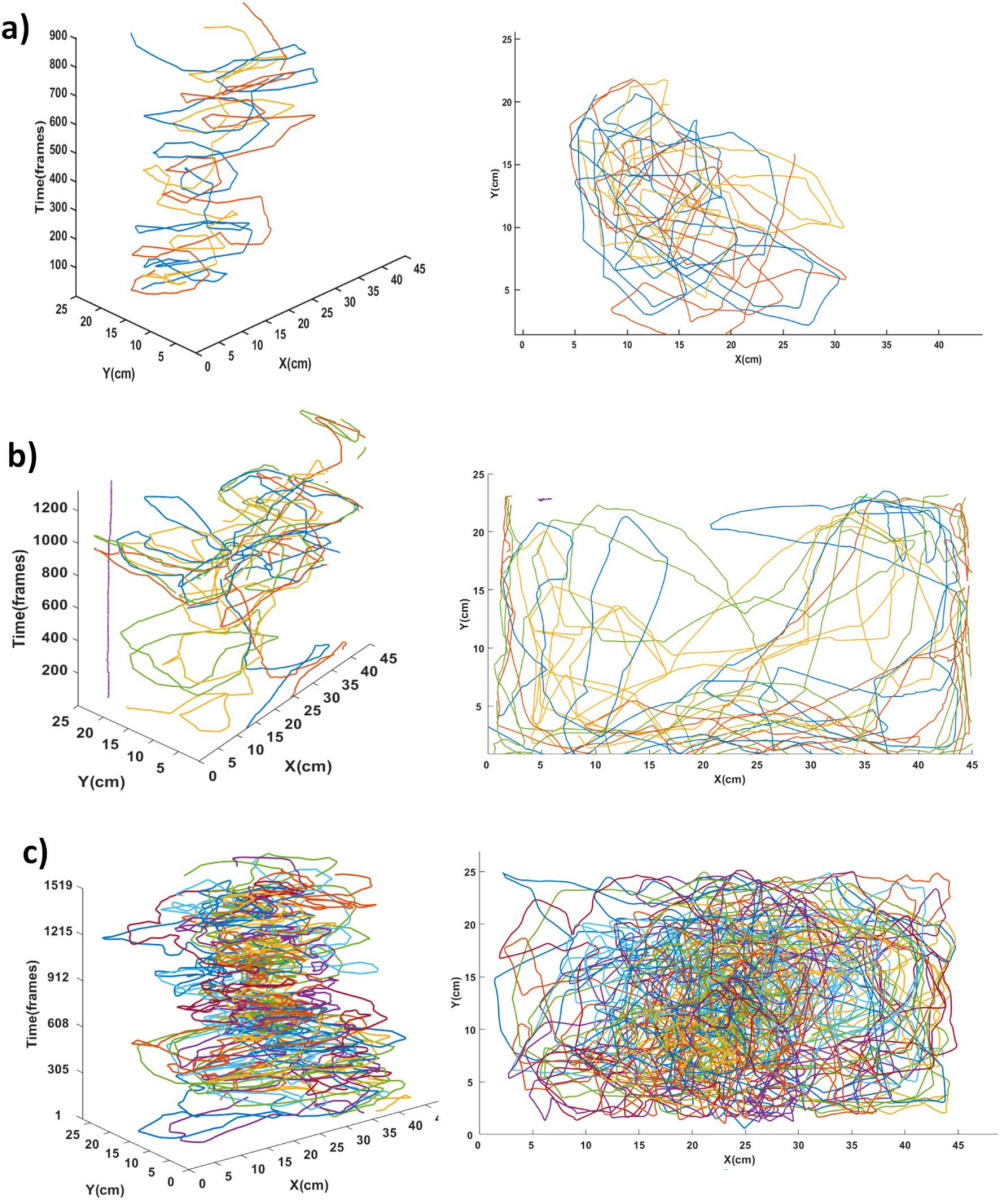
Result of tracking different groups of fish. **(a)** Tracking of group D1 with 3 fish and **(b)** group D2 with 6 fish, both in a state of slow motions, **(c)** group D6_food with 13 fish are in the feeding period (fast motions). The fish swim with greater agitation and agglomeration in the center of the aquarium, a space where the food was concentrated.

Figure 8 shows a visual example of tracking in three video sequences (D1, D2 and D6_food).

## Discussion

The method proposed in this work combined two techniques to detect and track fish schools: the YOLOv2 network and the Kalman filter. Previous tracking methods showed applications of fish swimming in shallow waters and slow motions^9,10,17,19,32^. Here, we test the proposed method in adverse situations such as the feeding period, which is widely used for training and conditioning fish. It was noticed that the quantity and agitation of the school of fish are important factors for the increase in occlusion, which makes detection difficult most of the time.

However, through this method, we showed that detection and tracking were obtained around 99% in low and high resolution images, with variation in the quantity of fish (up to 100 fish). High resolution images, such as the images available from idtracker.ai^2^, proved to be favorable for successful screening. In addition, it was observed that the spread of errors decreases when the fish have little occlusion.

In some methods, the fish's entire body segmentation is used to detect and identify the fish^9,10,17,32^, however we created a delimitation of the region of the fish head to facilitate detection, this approach has been presented in other works, but using different techniques^9,19^. The performance of the YOLOv2 network improves as training is increased, improving the result of the delimitation of the region of the fish head.

The fish was detected by calculating the centroid, based on the identification of the region of the fish head by YOLOv2. The Kalman filter was used to adjust the centroid, estimating the fish's position between frames, when there was loss of detection. A fish changes from one position to another quickly, and a method for estimating the position in the tank may fail to identify the fish's current position. An adjustment of the cost function has considerably improved the prediction of the state of the fish, making it possible to create trajectories. In order to establish the lost trajectories, the route was reconnected using the shortest distance. In this study, a much higher frame rate is not necessary, we used a frame rate considered low at 30 frames per second, and we were able to track the fish efficiently. Higher rates can compromise the algorithm's run time.

Although we were able to correctly identify fish at around 0.83 (CIR) in the feeding period, where fish can have rapid movements and many variations in the direction of movement, it is still a limitation when the number of occlusions between fish is high, as there may be an exchange of fish identification. In addition, long occlusions, which are greater than 15 frames, may have a greater chance of exchanging fish identification, due to the lack of an identification step.

Thus, the inclusion of an identification step, could improve the tracking of the fish in the entire route in the tank, especially after long occlusions, reducing the chances of propagating errors, compromising the performance of the method. The YOLOv2 network when trained with different classes can detect multiple objects from the same or different classes in the same image. When used as a unique identification method for several individuals of the same species in the same scene, a better performance is achieved in terms of speed and accuracy in carrying out the task. In idtracker.ai^2^, a fish identification step is used, without the need for an additional method to associate the detection of objects between frames, generating greater accuracy in tracking, especially during fish occlusions. A new approach could be attributed to our work, which includes a fish identification step to further improve tracking.

## Conclusion

In this paper, an effective method for detecting and tracking schools of fish swimming in calm and agitated behavior has been proposed. This method was satisfactory for detecting and tracking agglomerated and agitated fish during the feeding period. The method was based on the YOLOv2 network which delimits the region of the fish head so that the centroid can be calculated and, subsequently, the fish can be detected. The tracking was proposed by using the Kalman filter and adjusted by a cost function; in addition, the fragmentation of the trajectory has been reconnected to allow greater stability in the path of the fish. The method was evaluated in different schools and adverse situations, obtaining satisfactory results in all the metrics used for evaluation. However, including a fish identification step can further improve tracking in periods of many occlusions, preventing the spread of errors.

## Supplementary Information

Supplementary Information.
