# Supplementary material for: Zebrafish tracking using YOLOv2 and Kalman filter
Source: Sci Rep. 2021 Feb 5;11:3219. doi: 10.1038/s41598-021-81997-9 (PMC7865020; doi:10.1038/s41598-021-81997-9)
Supplement: Supplementary file 1 — Supplementary Information. [file 41598_2021_81997_MOESM1_ESM.docx]

**SUPPLEMENTARY INFORMATION**

**Zebrafish tracking using YOLOv2 and Kalman filter**

Marta de Oliveira Barreiros¹, Diego de Oliveira Dantas¹^,^², Luís Claudio de Oliveira Silva¹^,^², Sidarta Ribeiro³, Allan Kardec Barros¹

¹ Department of Electrical Engineering, Laboratory for Biological Information Processing (PIB), Federal University of Maranhão (UFMA), Av. dos Portugueses, 1966, Vila Bacanga, São Luís MA, 65080-805, Brazil.

² Department of Computational Engineering, Federal University of Maranhão (UFMA), Av. dos Portugueses, 1966, Vila Bacanga, São Luís, MA, Brazil.

³ Brain Institute, Federal University of Rio Grande do Norte (UFRN), Av. Sen. Salgado Filho, 3000 Candelária, Natal, RN, Brazil.

*Corresponding author: Marta de Oliveira Barreiros.

e-mail: marta-barreiros@hotmail.com
